# From escort to target, the multidimensional roles and prospects of platelets in tumor immune checkpoint inhibitor therapy

**DOI:** 10.3389/fimmu.2026.1764513

**Published:** 2026-04-24

**Authors:** Jiayu Xiao, Huaru Wang, Xinyue Liu, Zhiyuan Hu, Yingchun Xu, Xuzhen Qin

**Affiliations:** 1 Department of Clinical Laboratory, State Key Laboratory of Complex Severe and Rare Diseases, Peking Union Medical College Hospital, Chinese Academy of Medical Sciences & Peking Union Medical College, Beijing, China; 2 Chinese Academy of Sciences (CAS) Key Laboratory for Biomedical Effects of Nanomaterials and Nanosafety, Chinese Academy of Sciences (CAS) Center for Excellence in Nanoscience, National Center for Nanoscience and Technology, Beijing, China

**Keywords:** activation, engineering, ICIs, microenvironment, PD-L1, platelet, tumor

## Abstract

Immune checkpoint inhibitors (ICIs) have revolutionized cancer therapy by reinvigorating antitumor immunity through the blockade of inhibitory pathways such as programmed cell death protein 1 (PD-1)/programmed cell death protein ligand 1 (PD-L1) and cytotoxic T-lymphocyte-associated protein 4 (CTLA-4). Despite their remarkable clinical success, only a subset of patients derives durable benefit, whereas others exhibit primary or acquired resistance and develop immune-related adverse events (irAEs). These heterogeneous responses highlight an urgent need for robust biomarkers to predict therapeutic efficacy and for innovative combinatorial strategies to enhance clinical outcomes. Beyond their classical roles in hemostasis and thrombosis, platelets have recently emerged as pivotal modulators of tumor progression and immune regulation. Accumulating evidence indicates that platelets engage in dynamic crosstalk with tumor and immune cells, reshaping the tumor microenvironment (TME) and modulating the response to ICI therapy. Of note, platelet-associated immune checkpoint molecules (e.g., PD-L1) have shown great promise as liquid biopsy markers for patient stratification and real-time immunomonitoring. Furthermore, platelet-associated nucleic acids and traditional platelet parameters (such as platelet count and activation status) have been identified as accessible and effective biomarkers for predicting ICI responsiveness and irAEs. These platelet-derived components may also represent novel therapeutic targets to overcome resistance and potentiate ICI efficacy. Meanwhile, advances in biomaterials and genetic engineering have further enabled the development of platelet-based and platelet membrane (PM)-camouflaged delivery systems endowed with tumor-homing capacity, combinatorial drug delivery potential, and immune-responsive release properties. Collectively, these insights reposition platelets from passive participants to active regulators and versatile therapeutic platforms in cancer immunotherapy, providing a conceptual foundation for next-generation platelet-guided precision immunotherapeutic strategies.

## 1 Introduction

The revolution in tumor immunotherapy began with the discovery and application of immune checkpoints. In the 1990s, James Allison and Tasuku Honjo identified the key immunoregulatory functions of cytotoxic T-lymphocyte-associated protein 4 (CTLA-4) and programmed cell death protein 1 (PD-1), respectively, thus ushering in a new era of tumor therapy centered on Immune checkpoint inhibitors (ICIs) (1–3). Clinical practice based on such drugs has significantly improved the survival prognosis of patients with a wide range of tumors, with some patients even achieving long-term remission (4–7). Both scientists were also awarded the Nobel Prize in Physiology or Medicine in 2018 for this breakthrough.

However, since the tumor and the immune cells in the tumor microenvironment (TME) are in a process of continuous interaction and dynamic changes during tumor progression (8–10), the application of ICIs in clinical antitumor therapy still faces many challenges: only a fraction of patients benefit from them (11) and a substantial number of patients develop acquired resistance (12), the high incidence of immune-related adverse events (irAEs) (13), etc. Currently, programmed cell death protein ligand 1 (PD-L1) detection by immunohistochemistry (IHC) is widely employed in clinical practice to guide ICI therapy (14, 15). However, the reliability of this assay is constrained by the spatial heterogeneity of tumors (16, 17). In addition, histologic assessments are inherently limited in their ability to support longitudinal monitoring, despite the fact that PD-L1 expression is dynamically regulated by various factors, including cytokines (18), microRNAs (miRNAs) (19), oncogenes (20), and nucleoplasmin (21). PD-L1 levels may also fluctuate in response to treatments such as radiotherapy and chemotherapy (15, 22–24). Notably, several clinical trials have reported that a subset of patients with undetectable PD-L1 expression still derived clinical benefit from ICIs (25–27). These limitations underscore the urgent need to identify novel biomarkers capable of accurately reflecting the tumor immune microenvironment and capturing its dynamic evolution over time.

In recent years, liquid biopsy, represented by circulating tumor DNA (ctDNA), circulating tumor cells (CTCs), exosomes, and tumor-educated platelets (TEPs), has heralded a transformative era in cancer diagnosis and management (28). Among these components, tumor-educated platelets not only protect circulating tumor cells from immune-mediated clearance but also facilitate their distant metastasis (29, 30). Furthermore, TEPs function as potential transit stations for exosomes and ctDNA (31, 32), thereby playing an indispensable role in liquid biopsy. This blood-based testing offers a promising minimally invasive, safe, and sensitive alternative or complement to traditional tissue biopsy.

Notably, platelets, which were traditionally regarded as central mediators of hemostasis and thrombosis, have increasingly been recognized as active regulators of immune responses within the TME (33). As the second most abundant cellular component in peripheral blood (34), platelets exhibit remarkable functional versatility, contributing not only to vascular integrity but also to key oncogenic processes including tumor angiogenesis, metastasis, and immune evasion (35, 36). Beyond these classical roles, accumulating evidence indicates that platelets engage in intricate crosstalk with both tumor and immune cells through direct contact or the release of bioactive molecules (37), thereby influencing antitumor immunity and disease progression.

Platelets are known to express and transfer a range of immunomodulatory molecules, including PD-L1, which can attenuate effector T-cell activity and promote immune tolerance (38). Their unique biological features, including small size, high abundance, dynamic circulation, and efficient infiltration into tumor parenchyma, enable them to interact intimately with the TME, positioning platelets as emerging players in cancer immunotherapy research (39). The presence of platelet-associated immune checkpoint molecules may also provide novel mechanistic insights into ICI resistance and highlights the immunoregulatory potential of these anucleate cells. Moreover, many clinical and translational studies have revealed that platelet-associated nucleic acids, platelet count and activation status also correlate with therapeutic responses and irAEs in patients receiving ICIs therapy (40–42). These findings further underscore the active involvement of platelets in sculpting the immune landscape and thereby affecting immunotherapy outcomes. In parallel, advances in biomaterials and genetic engineering have facilitated the design of platelet-based and platelet membrane (PM) coated drug delivery systems (43). By exploiting their inherent tumor-homing properties and immunoregulatory potential, platelets are transformed from drivers of tumor immunosuppression to intelligent immunotherapy delivery systems. This paradigm shift may break through the efficacy bottleneck of current ICI therapies, potentially catalyzing disruptive innovations in cancer immunotherapy.

**Figure 1:**
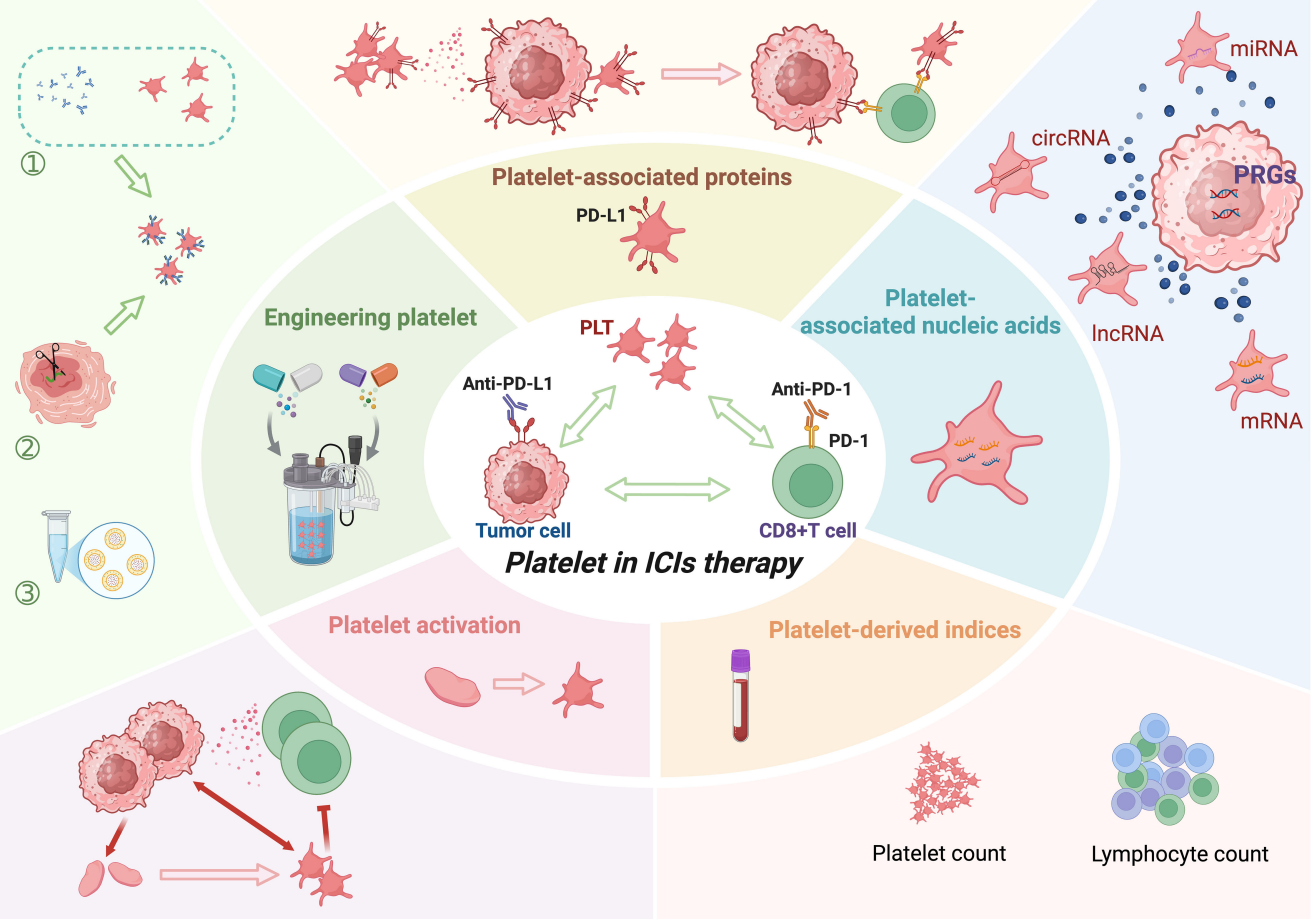
The multifaceted role and applications of platelets in tumor immune checkpoint inhibitor therapy. Created in https://BioRender.com.

This review provides a comprehensive overview of the multifaceted roles of platelets and their derivatives in modulating tumor immunity and influencing ICI efficacy, as illustrated in **Figure 1**. We particularly focus on platelet-associated immune checkpoint molecules as emerging biomarkers and therapeutic targets. Furthermore, we examine the mechanistic pathways by which platelets influence the tumor immune microenvironment, emphasizing their evolving role from “immune escorts” to potential “therapeutic targets.” By integrating the latest experimental and clinical evidence, this review aims to provide new conceptual frameworks and translational perspectives for leveraging platelet biology to optimize individualized cancer immunotherapy.

## 2 Platelet-associated proteins as regulators and biomarkers of immune checkpoint modulation

Increasing evidence indicates that platelet-associated proteins, encompassing membrane proteins, signaling proteins, enzymes, and transcription factors, actively participate in shaping tumor immune crosstalk and influencing the therapeutic outcomes of ICI therapy. These proteins are either synthesized endogenously by the platelets or derived from other cellular sources, including tumor cells (44). Platelets activated by the TME release these proteins, playing a pivotal role in modulating the tumor immune microenvironment, which in turn influences cancer progression and metastasis. The interaction between platelets and tumor cells is complex and multifaceted, involving several signaling pathways and molecular interactions. For example, platelets activate the FAK/PI3K/AKT signaling cascade by upregulating the expression of integrin-linked kinase and integrin beta-3, thereby contributing to the progression of intrahepatic cholangiocarcinoma (45). Furthermore, platelet activation also regulates the release of angiogenic proteins, such as vascular endothelial growth factor (VEGF) and transforming growth factor-beta (TGF-β), which are crucial for tumor angiogenesis (46).

Compared with the complexity and high cost of biomolecular detection on tumor cells, the detection of platelet-associated proteins offers a more practical and minimally invasive approach for clinical application, allowing dynamic and longitudinal monitoring of immune responses during ICI treatment. Furthermore, therapeutic strategies targeting these platelet-derived proteins or their signaling pathways may provide novel opportunities to potentiate antitumor immunity while mitigating irAEs.

### 2.1 Platelet-mediated regulation of tumor cell immune checkpoint molecule expression

**Figure 2:**
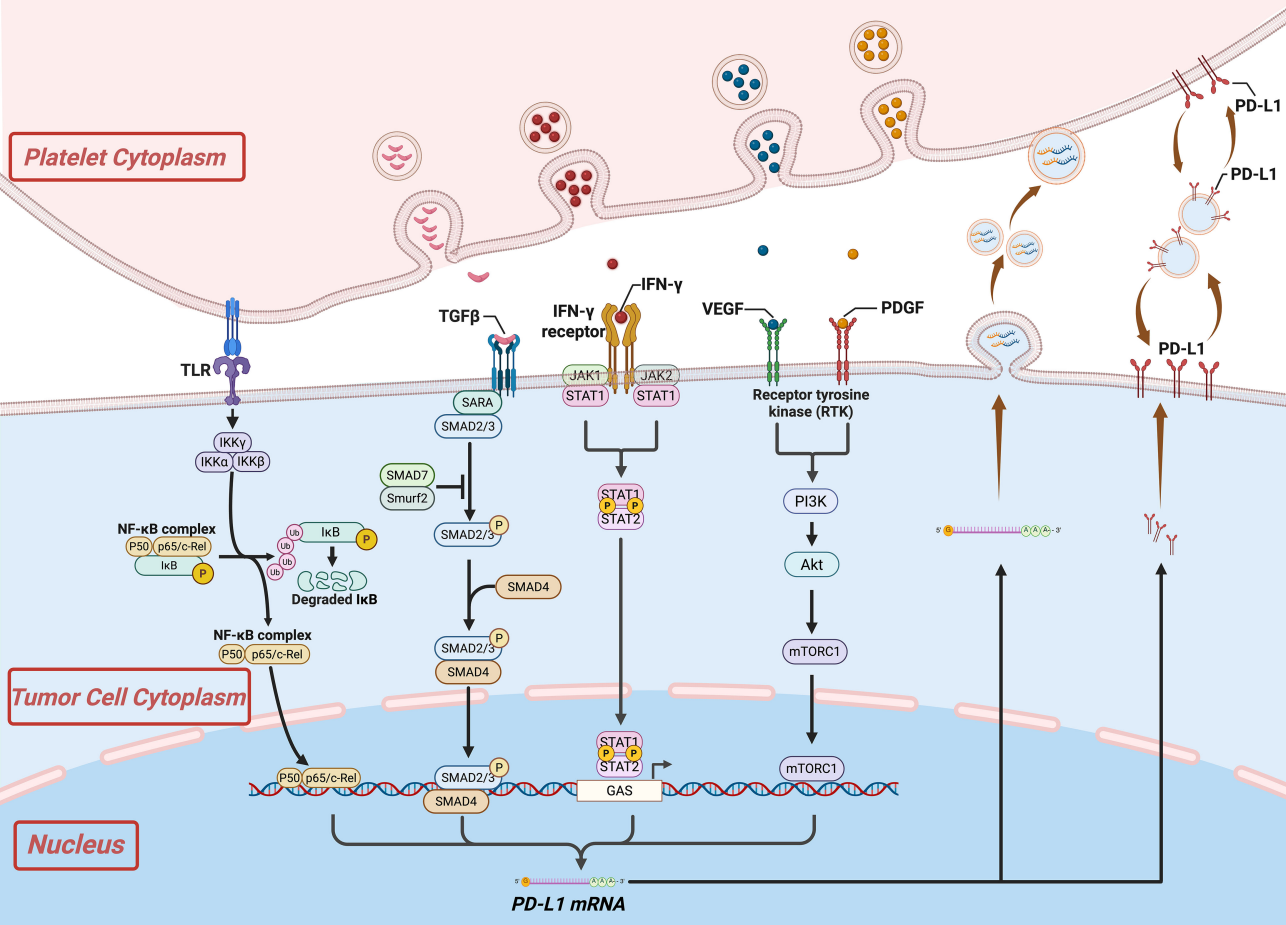
Interactions between platelets and tumor cells promote the production and transfer of immune checkpoint molecules. Created in https://BioRender.com.

In clinical practice, tumor tissue PD-L1 expression is commonly used as a predictive biomarker for ICIs efficacy (47). However, as mentioned earlier, its predictive performance remains suboptimal (48). As an important immunemodulatory component within the TME, platelets can secrete a variety of bioactive mediators and engage in direct or indirect interactions with tumor cells, thereby influencing immune regulation, including modulation of PD-L1 expression on tumor cells, as illustrated in **Figure 2**.

Notably, previous studies have found that platelets are more efficient at transferring structural components such as lipids, proteins, and RNA to tumor cells than tumor cells are at transferring them to platelets, whether through direct contact, internalization or via extracellular vesicles (49). This process educates the tumor cells to acquire a highly dynamic and invasive phenotype. For instance, platelet-derived serotonin increases PD-L1 expression in tumor cells through serotonylation, a covalent modification characterized by the formation of an amide bond between serotonin and glutamine residues, which activates small GTPase. This process concurrently reduces the population of CD8+ tumor-infiltrating T cells (50). The enzyme tryptophan hydroxylase (TPH), which is encoded by the genes TPH1 and TPH2, serves as the rate-limiting factor in the biosynthesis of serotonin, thereby playing a pivotal role in this biochemical process (51). Previous studies have demonstrated that pharmacological inhibition of TPH1 using Telotristat reduces serotonin levels and augments the antitumor efficacy of ICIs (50).

Additional evidence indicates that platelets can also upregulate tumor PD-L1 expression via multiple signaling pathways, including NF-κB, TGFβR1/Smad, and EGFigure cascades (52–54), thereby enriching the mechanistic understanding of platelet-mediated PD-L1 regulation. Furthermore, in addition to PD-L1, platelets can also upregulate the expression of CD155 on CTCs, which interacts with the immune receptor TIGIT on NK cells, thereby inhibiting NK cell-mediated cytotoxicity and facilitating immune evasion (55).

### 2.2 Immune checkpoint molecules expressed on platelets

Recent findings have revealed that, in addition to tumor cells, PD-L1 is also expressed on circulating platelets. A significant proportion of tumor patients exhibit an increased frequency of PD-L1+ platelets, and similar elevations have been observed among smokers compared to healthy individuals (56). Interestingly, in patients receiving anti-PD-L1 immunotherapy, PD-L1 expression on platelets transiently decreases without significant changes in platelet count (57), suggesting that platelet PD-L1 bears a high degree of structural similarity to tumor cell PD-L1 and possesses the functional potential to engage PD-1 on T lymphocytes and suppress immune activation.

Hinterleitner et al. demonstrated that tumor cell surface PD-L1 can be transferred to platelets through a fibronectin-1/integrin α5β1/GPIbα-dependent mechanism via direct cell-cell contact. Clinically, high platelet PD-L1 expression correlates with shorter progression-free survival (PFS) in patients receiving non-ICI therapies but with enhanced responsiveness to ICIs (38, 58), implicating its dual role in immune evasion and therapeutic susceptibility. Zhu et al. further confirmed PD-L1 transfer from tumor cells to platelets using nanoprobes (59).

Remarkably, some patients exhibit PD-L1-negative results in tumor biopsy samples yet display PD-L1+ platelets in circulation. These patients also responded well to ICI therapy (60). Two mechanisms may underlie this phenomenon. First, platelet PD-L1 can functionally bind PD-1 to suppress T-cell cytotoxicity, providing a plausible explanation for why some PD-L1-negative tumors still benefit from anti-PD-L1 therapy. Second, tumor PD-L1 expression is highly heterogeneous, and biopsy-based assessment may not reliably reflect overall PD-L1 status, contributing to the limited correlation between PD-L1 expression and objective response rate (ORR) (61).

Moreover, clinical studies indicate that platelet-associated PD-L1 mRNA levels correlate significantly with PFS, whereas tumor tissue PD-L1 expression does not (40), suggesting that platelet PD-L1 may have an origin beyond simple tumor-cell transfer. Supporting this hypothesis, Zaslavsky et al. found that PD-L1-negative tumor cells acquire PD-L1 positivity after co-culture with PD-L1+ platelets (60). Collectively, these findings indicate a bidirectional PD-L1 transfer between tumor cells and platelets. Given the close association between platelet PD-L1 and the immune landscape, it holds promise as a dynamic biomarker for predicting and monitoring ICI responsiveness.

### 2.3 Immunomodulatory roles of platelet-associated immune checkpoint molecules

As described above, platelet PD-L1 can indeed engage PD-1 on T lymphocytes, leading to suppression of T-cell activation and protection of tumor cells from immune attack (38). Similar immunosuppressive mechanisms have been observed in COVID-19 patients, where platelet PD-L1 suppresses CD4+ T-cell function and contributes to disease progression (62). Furthermore, platelet PD-L1 activates the AKT signaling pathway to inhibit platelet apoptosis, which may partly explain the severe thrombocytopenia observed in some cancer patients undergoing ICI therapy (63). Given the substantial structural and functional similarities between PD-L1 on platelets and tumor cells, further research is need warranted to determine whether the dosage of ICIs should be adjusted based on the levels of PD-L1+ platelets in peripheral blood.

In melanoma, PD-L1-expressing exosomes have been detected in circulation; these vesicles inhibit CD8+ T-cell cytotoxicity and are markedly elevated during early immunotherapy (64). Subsequent studies suggest that these PD-L1+ exosomes may originate from platelets or interact closely with them (65), indicating that platelet PD-L1 contributes to immune modulation not only via direct cell-cell contact but also through extracellular vesicle-mediated communication.

Beyond PD-L1/PD-1, emerging evidence points to additional immune checkpoint interactions involving platelets, such as the HLA-E:CD94-NKG2A axis. This pathway plays a critical role in enabling CTCs to evade NK cell-mediated clearance. Platelet-derived regulator of G-protein signaling 18 (RGS18) promotes HLA-E expression through the AKT-GSK3β-CREB signaling cascade, thereby protecting CTCs and facilitating hematogenous metastasis (66). In addition, platelets can interact directly with immune cells, modulating their functions and contributing to immune responses. For instance, platelet-neutrophil interactions enhance the formation of neutrophil extracellular traps, which play a significant role in host defense and inflammation (67). CTCs-released high-mobility group box1 (HMGB1) interacts with TLR4 on platelets and mediates platelet-tumor cell interaction (68). TLR4 activates platelets in an ERK5- and GPIIb/IIIa-integrin-dependent manner, causing them to form aggregates with CTCs. This interaction facilitates the capture of these CTCs by neutrophil extracellular traps and subsequently promotes their distant metastasis (69). Platelets also interact with regulatory T cells, modulating their proliferation and function, which can further influence immune responses and cancer progression (70). Such coordinated crosstalk among multiple immune checkpoint pathways may also underlie the development of clinical resistance to ICI therapy.

In summary, accumulating evidence highlights the pivotal role of platelets in regulating tumor immune evasion through immune checkpoint pathways. Platelets actively participate in shaping the tumor immune microenvironment by modulating PD-L1 expression on tumor cells and expressing functional immune checkpoint ligands themselves. The discovery that PD-L1 can be transferred bidirectionally between tumor cells and platelets not only deepens our understanding of platelet-tumor crosstalk but also introduces platelet PD-L1 as a promising liquid biomarker for predicting and dynamically monitoring the efficacy of ICI therapy. Furthermore, platelet-derived PD-L1 and other immune checkpoint molecules such as HLA-E engage in complex regulatory networks involving NF-κB, TGFβ/Smad, EGFR, and AKT-GSK3β-CREB signaling cascades, contributing to immune suppression and metastasis. These findings suggest that platelet-associated immune checkpoint molecules may serve as both indicators and mediators of therapeutic response and resistance. Future research focusing on elucidating their precise cellular origins, regulatory mechanisms, and clinical implications could pave the way for novel platelet-targeted immunomodulatory strategies that enhance the efficacy and safety of cancer immunotherapy.

## 3 Platelet-associated nucleic acids and platelet-related genes: emerging regulators and biomarkers in ICI therapy

Beyond their protein-based immunomodulatory roles, platelets also serve as dynamic reservoirs and carriers of diverse nucleic acids, including messenger RNAs (mRNAs), miRNAs, circular RNAs (circRNAs), and long noncoding RNAs (lncRNAs) (71). These molecules enable platelets to engage in bidirectional molecular communication with tumor and immune cells, thereby influencing tumor progression and therapeutic response. Platelets have emerged as dynamic entities capable of interacting with tumor cells and modulating their environment through RNA transfer. This process is facilitated by platelet-derived microparticles (PMPs), which infiltrate solid tumors and transfer platelet-derived RNAs, including miRNAs, to tumor cells. This transfer can induce tumor cell apoptosis and inhibit tumor growth, as demonstrated by the significant role of miR-24 in regulating mitochondrial dysfunction and tumor progression (72). Tumor cells can also transfer mutant RNAs into platelets, altering their RNA profile and creating a distinct RNA signature that can serve as a biomarker for cancer diagnosis and monitoring. Meanwhile, these tumor-derived genetic materials can be processed, and functionally utilized by platelets (73), which may reflect and even modulate the immune status of the host. This dynamic exchange of RNA not only highlights the potential of platelets as a liquid biopsy biosource but also underscores their role in supporting tumor growth and dissemination by modulating the tumor microenvironment (74). In parallel with these findings, platelet-related genes (PRGs) involved in platelet activation, aggregation, and signaling transduction have been shown to correlate with prognosis and responsiveness to ICIs. Together, these discoveries highlight the dual mechanistic and predictive significance of platelet-associated nucleic acids and PRGs, providing new opportunities for developing platelet-based biomarkers and therapeutic targets to enhance immunotherapy efficacy.

### 3.1 Platelet-associated nucleic acids and prognostic significance

As mentioned previously, platelets, being megakaryocytic fragments, lack a nucleus and DNA available for RNA transcription, thus requiring RNA transcripts derived from megakaryocytes to maintain their function repertoire (75–77). Similarly, it has been demonstrated that tumor cells are capable of exporting various RNA species through multiple mechanisms, including microvesicle encapsulation (78), copurification with the Argonaute 2 ribonucleoprotein complex (79), and binding to high-density lipoprotein (HDL) (80). Platelets, in turn, are able to pick up these RNAs (73), where they are processed for pre-mRNA splicing, pre-miRNA processing, and mRNA translation (81–83), as well as transport them to distant tissues and organs (84). A recent investigation into PD-L1 mRNA derived from TEPs demonstrated that TEP-associated PD-L1 mRNA levels were not significantly correlated with tumor PD-L1 tumor proportion scores (TPS), and even exhibited a trend toward negative association. Despite this lack of concordance with tumor PD-L1 expression, TEP-derived PD-L1 mRNA showed robust prognostic and predictive relevance in the context of ICI therapy (40). Patients with high TEP-PD-L1 mRNA levels displayed a markedly greater likelihood of responding to immunotherapy, with higher rates of partial response (PR) compared with those in the low-expression group (44.4% vs. 13.9%). Notably, even among TPS-negative patients, elevated TEP-PD-L1 mRNA levels were associated with significantly prolonged median PFS relative to their low-expression counterparts. These findings underscore the superior and TPS-independent predictive value of platelet-derived PD-L1 mRNA as a non-invasive biomarker for guiding ICI treatment.

Aside presence of mRNAs, platelets contain other classes of RNA molecules, such as miRNAs, circRNAs, and lncRNAs (71). These RNAs participate in the regulation of immune and oncogenic pathways, reflecting the dynamic communication between the TME and systemic immunity. Nevertheless, the specific mechanisms through which tumor-derived signals regulate RNA splicing and translation within platelets remain largely unexplored.

Recent evidence also suggests that platelets can capture and preserve free fetal DNA deriving from tumor cells. This phenomenon not only advances early cancer detection but also provides mechanistic insights into platelet-mediated tumor promotion. Such nucleic acid-based interactions hold potential for refining predictive biomarkers and improving the efficacy of ICI therapy through combination strategies involving platelet-targeting agents (32).

### 3.2 PRGs and prognostic significance

In addition to these nucleic acids in platelets, PRGs involved in platelet activation, aggregation, and signal transduction are likewise inextricably linked to tumor ICI treatment prognosis. Transcriptomic analyses integrating PRG expression profiles with clinical data from immunotherapy-treated patients have facilitated the identification of key PRGs and the establishment of predictive models for ICI therapeutic efficacy and prognosis.

Li et al. reported that activation of platelets in contact with hepatocellular carcinoma cells significantly upregulated the expression of genes such as PRKCD, HRAS, TUBA4A, EGF, GNG4, CFL1, PPIA, GNA12, OLA1, and ANXA5, among which PRKCD was found to play a pivotal role in both the malignant phenotype of tumors and platelet activation, acting as a central mediator in tumor progression (85). Moreover, several studies have developed platelet risk scores (PRS) derived from PRG signatures to predict ICI treatment outcomes in common malignancies, including lung squamous carcinoma, hepatocellular carcinoma, and bladder cancer (86–88).

Functional studies further demonstrated that knockdown of the alpha1C-tubulin (TUBA1C), which is a significantly upregulated PRG in bladder cancer patients, could significantly inhibit tumor proliferation (89). These findings reveal the potential role of TUBA1C in tumor growth and provide a new strategy for targeted therapy.

With the continuous development of highly sensitive and specific detection technologies such as high-throughput sequencing, more and more platelet-related nucleic acid sequences involved in regulating the tumor immune microenvironment are being uncovered. Not only can we predict the prognosis of tumor immunotherapy by detecting these PRGs, but we can also study their physiological roles and pathogenic mechanisms, and carry out targeted and precise treatments to regulate the tumor immune microenvironment without affecting the normal physiological functions of platelets, which is expected to ultimately enhance the efficacy of ICI therapy. Nonetheless, the majority of contemporary research studies are predicated on validation within small-sample cohorts. Considering the substantial heterogeneity present across various tumors and populations, as well as the diverse array of detection methodologies for these biomarkers, most identified biomarkers remain at the preliminary screening stage. Future endeavors must focus on conducting multicenter, large-scale validation studies of these initially screened biomarkers and on developing standardized testing protocols. Such efforts are crucial to overcoming technical challenges, including platelet detection instability, RNA degradation and contamination, thereby enhancing the reproducibility of test results. This is imperative for ensuring the dependable application of these biomarkers in clinical settings. Moreover, our current comprehension of the mechanisms of action of these nucleic acids and their relationship with immune checkpoint signaling pathways is only the tip of the iceberg regarding their role in regulating tumor cell immune evasion. The integration of comprehensive genomic and proteomic analyses to further investigate the immune regulatory mechanisms of these novel biomarkers will significantly augment their translational medical value.

## 4 Platelet count and platelet-derived indices as predictive biomarkers in ICI therapy

Building on the molecular insights derived from platelet-associated nucleic acids and PRG signatures, recent research has revealed that tumor-driven transcriptional and post-transcriptional reprogramming in platelets can reshape their immunemodulatory functions and influence therapeutic outcomes in ICI therapy. However, despite the mechanistic depth of these findings, their clinical translation remains limited by technical complexity and cost constraints. To bridge this gap, increasing attention has turned toward peripheral platelet-derived indices that are easily obtainable, cost-effective, and dynamically reflect the crosstalk between platelets and immune responses. Among these parameters, platelet count and platelet-based ratios, particularly the platelet-to-lymphocyte ratio (PLR), have emerged as promising biomarkers for predicting both treatment efficacy and irAEs in patients receiving ICI therapy.

Platelets are anucleate cytoplasmic fragments derived from megakaryocytes, with a short lifespan of approximately 7–10 days, necessitating the daily production of nearly 100 billion platelets in adults to maintain homeostasis (90). In certain solid tumors, elevated platelet counts (thrombocytosis) have been frequently observed (91). Notably, variations in platelet count have been associated with differential responses and outcomes in patients undergoing ICI therapy. Patients with lower baseline platelet counts tend to exhibit longer PFS and overall survival (OS) following ICI treatment compared with those presenting higher counts (92), highlighting platelet count as a readily accessible biomarker for immunotherapy efficacy prediction.

A retrospective study of patients with malignant mesothelioma treated with pembrolizumab revealed that individuals with pretreatment platelet counts ≤400 × 10^9^/L achieved significantly longer PFS and OS (93). Moreover, dynamic changes in platelet count during treatment also correlate with prognosis. Patients who developed mild thrombocytopenia (70-150×10³/μL) during ICI therapy had markedly improved OS compared to those who did not (94).

Beyond absolute platelet numbers, composite hematologic indices integrating platelet counts with other immune-inflammatory parameters (such as neutrophil, monocyte, and lymphocyte counts) have demonstrated even greater predictive power. For instance, the pan-immune-inflammation value has shown strong prognostic relevance for OS and PFS in patients receiving ICIs (95). Among these, the platelet-to-lymphocyte ratio (PLR) has emerged as one of the most robust and widely used markers due to its accessibility and reproducibility. Elevated pre-treatment PLR has been consistently linked to poorer survival outcomes across multiple cancers. In metastatic melanoma, a higher baseline PLR and a greater increase in platelet count during therapy were associated with shorter OS and PFS (96), findings that were further corroborated in non-small cell lung cancer (NSCLC) patients treated with nivolumab, where elevated pre-treatment PLR predicted a lower response rate and reduced OS and PFS (41). Subsequent investigations extended these observations to melanoma, nasopharyngeal carcinoma, gastric cancer, and recurrent glioblastoma, confirming PLR as a reliable predictor of immunotherapy efficacy across tumor types (97–100).

In addition to efficacy prediction, platelet counts and related indices also inform the risk of irAEs. Low platelet counts have been associated with increased susceptibility to cytokine release syndrome and ICI-related pneumonitis (101, 102). Similarly, patients with low baseline PLR exhibit a significantly higher incidence of irAEs (103–105). Interestingly, clinical studies have shown that the occurrence of irAEs often correlates with better treatment outcomes (106–108), a pattern that aligns with PLR-based predictive analyses (41). Furthermore, thrombocytopenia itself can represent an irAE (109–111), likely driven by the production of autoantibodies against platelets following ICI exposure (112) or with the inhibition of signaling pathways that suppress platelet apoptosis due to PD-L1-mediated activation of the AKT pathway (63). Nevertheless, the incidence of thrombocytopenia among patients undergoing immunotherapy is relatively low (0.2%–2.8%), and most cases are neither severe nor fatal (113, 114). This immune-mediated platelet reduction may reflect a heightened systemic immune activation, potentially explaining the improved PFS and OS observed in patients who develop mild thrombocytopenia without severe hematologic compromise.

Collectively, platelet count and derived indices, particularly PLR, emerge as potent, cost-effective, and noninvasive predictors of both immunotherapy efficacy and irAE risk. The simplicity of their measurement and their capacity for real-time longitudinal monitoring provide distinct advantages over conventional tissue-based biomarkers, such as PD-L1 IHC. These characteristics position platelet-based parameters as promising tools for precision immunotherapy optimization and personalized treatment decisions in the clinical management of cancer patients undergoing ICI therapy.

## 5 Platelet activation: a central modulator of tumor immunity and response to ICIs

**Figure 3:**
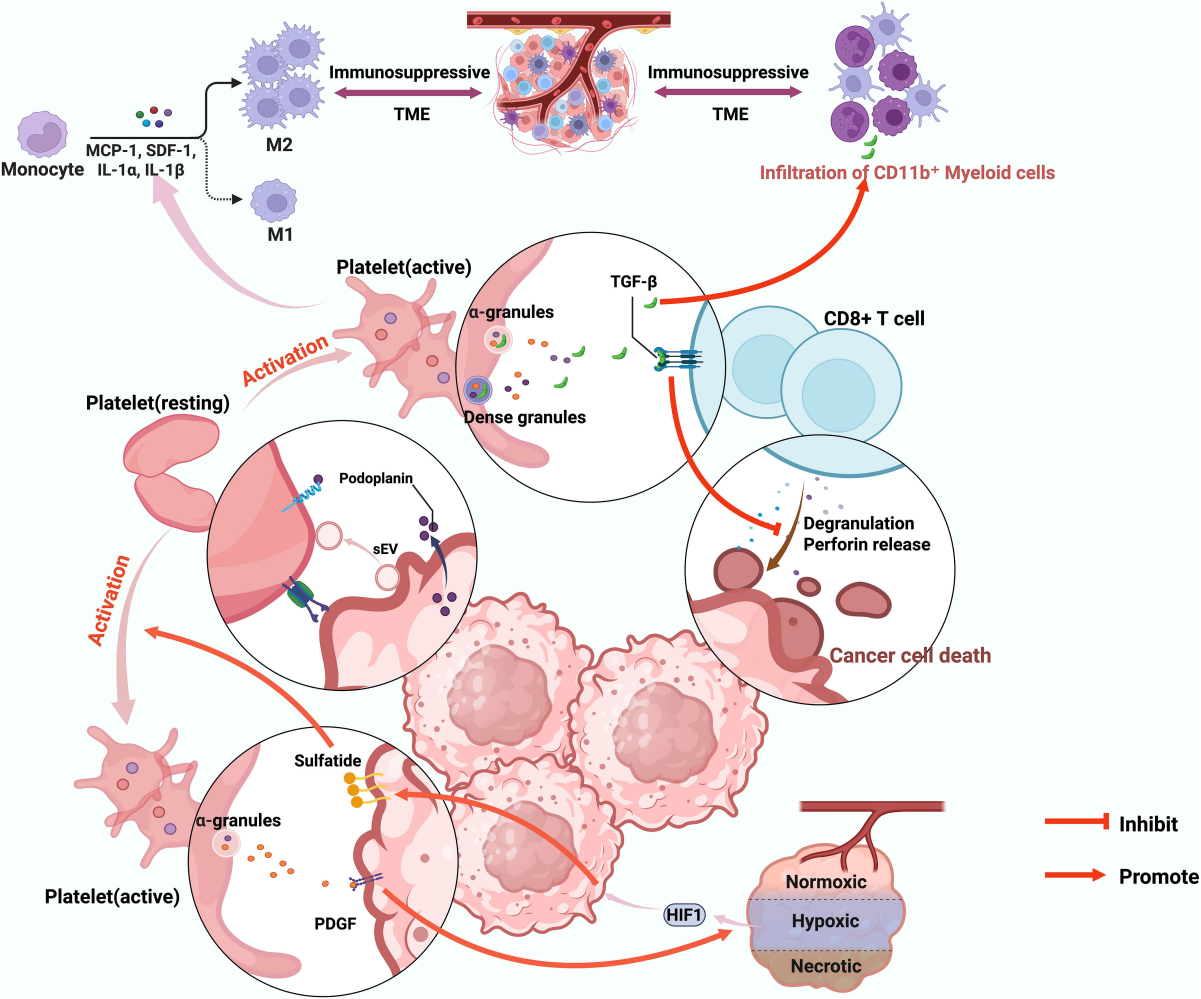
Platelet activation following interaction with tumor cells promotes an immunosuppressive tumor microenvironment. Created in https://BioRender.com.

While platelet count and derived indices provide indirect clinical insights into immune modulation, the functional activation state of platelets offers a more mechanistic perspective on their role in shaping antitumor immunity and influencing ICI responses, as illustrated in **Figure 3**. Platelet activation is a tightly regulated process central to thrombosis and hemostasis. However, within the oncological context, it transcends its traditional role in vascular repair, emerging as a pivotal contributor to tumor progression, immune suppression, and resistance to therapy.

### 5.1 Tumor-driven platelet activation and its consequences

Emerging evidence indicates that tumor cells can directly stimulate platelet activation through cell-cell contact or by secreting signaling mediators such as small extracellular vesicles (sEVs) and podoplanin (31, 115, 116). Once activated, platelets release a diverse array of bioactive molecules from their alpha and dense granules, thereby stabilizing the tumor vasculature and preventing hemorrhage within the TME (117). However, this hemostatic stabilization comes at an immunologic cost, activated platelets secrete multiple cytokines and growth factors that remodel the TME toward an immunosuppressive phenotype.

Among these mediators, platelet-derived TGF-β has been identified as a potent suppressor of antitumor immunity. As mentioned earlier, TGF-β released from activated platelets not only promotes tumor angiogenesis but also impairs bispecific antibody-mediated cytotoxicity by reducing T cell degranulation and perforin release, thereby attenuating the lysis of tumor targets (118). Sustained activation of the TGF-β signaling pathway also diminishes both the frequency and effector function of CD8+ T cells while promoting the infiltration of CD11b+ myeloid cells, ultimately driving immune evasion and resistance to ICI therapy (119). Moreover, platelet-derived TGF-β and direct platelet-tumor cell contacts synergistically activate the TGF-β/Smad and NF-κB pathways, leading to an invasive mesenchymal-like phenotype in cancer cells (30). Concurrently, chimeric extracellular vesicles generated by the interaction between platelets and tumor cells can also promote epithelial-mesenchymal transition (EMT) in CTCs and activate endothelial (120). Moreover, previous studies have demonstrated at the genetic level, through *in vitro* co-culture of platelets and CTCs, that platelets can promote the maintenance of a partial EMT state in CTCs through indirect contact, thereby enhancing their plasticity (121). This EMT is essential for metastasis, as it enhances the invasive capabilities and facilitating extravasation of tumor cells.

TEPs further potentiate this immunosuppressive cascade by secreting a spectrum of chemokines, including macrophage chemotactic protein-1 (MCP-1), interleukin-1α (IL-1α), IL-1β, and stromal cell-derived factor-1 (SDF-1), which collectively recruit circulating monocytes to the tumor niche and induce their polarization into tumor-associated macrophages of the M2-like phenotype (122). This crosstalk establishes a feedback circuit that reinforces the immunosuppressive TME and contributes to therapeutic refractoriness.

### 5.2 Molecular mechanisms linking hypoxia and platelet activation

Intratumoral hypoxia, a characteristic feature of advanced malignancies, further exacerbates platelet activation. Under hypoxic conditions, the transcription factor hypoxia-inducible factor 1 (HIF-1) initiates the galactosylceramide sulfotransferase-sulfatide signaling axis, leading to upregulation of sulfatide expression on surfaces of tumor cells (123). This modification strengthens platelet-tumor cell interactions, promotes platelet activation, and simultaneously shields tumor cells from immune-mediated cytotoxicity (124, 125). Activated platelets not only protect CTCs from shear stress-induced damage in the bloodstream and facilitate their evasion of immune surveillance (29), but also transfer platelet-derived normal major histocompatibility complex (MHC) class I onto the tumor cell surface, enabling escape from T-cell-mediated immunity without inducing susceptibility to NK cell reactivity (126). Furthermore, researchers have used computational modeling at the cellular scale to demonstrate that activated platelets adhering to CTCs help them actively arrest at the endothelium, thereby promoting distant metastasis (127). Additionally, activated platelets can release platelet-derived growth factors (PDGFs) stored in alpha granules (128, 129). These PDGFs can activate the Ras/MAPK and PI3K/AKT signaling pathways in tumor cells, enhancing HIF-1 stability and transcriptional activity in a hypoxia-independent manner (130). Such reciprocal reinforcement between platelet activation and HIF-1 signaling represents a self-perpetuating circuit contributing to immune evasion and ICI resistance. Furthermore, PDGFB plays a significant role in recruiting cancer-associated fibroblasts and remodeling the extracellular matrix, thereby promoting a malignant transformation of the tumor microenvironment (131).

Moreover, sulfatide has been implicated in promoting the formation of platelet-leukocyte aggregates (124), which are not only play a pivotal role in arterial thrombogenesis (132), but also serve as potential biomarkers for irAEs during ICIs therapy. For instance, an elevated proportion of platelet-CD4+ T cell complexes in peripheral blood has been linked to an increased risk of skin-associated irAEs, while correlating with a lower incidence of grade ≥2 systemic irAEs. Conversely, a lower proportion of these complexes is linked to a greater risk of non-skin-associated irAEs and a higher likelihood of experiencing grade ≥3 severe irAEs (37). These observations highlight the dual role of platelet activation, serving both as a mediator of immune modulation and as a potential marker of immune dysregulation during ICI treatment.

### 5.3 Targeting platelet activation for therapeutic benefit

Given its multifaceted roles, targeting platelet activation has emerged as a promising therapeutic strategy to enhance ICI efficacy. Zhou et al. developed an albumin-based perfluorotributylamine nanoparticles (PFTBA@Alb) designed to inhibit platelet activation and disrupt tumor vascular barriers, thereby promoting intratumoral T-cell infiltration. When combined with ICIs, this approach significantly improved antitumor efficacy (133). Similarly, therapeutic strategies aimed at inhibiting platelet-derived factors responsible for vascular stabilization could potentiate the destruction of tumor vasculature, thereby facilitating immune cell infiltration and amplifying the ICI efficacy (117). In addition, innovative therapeutic strategies targeting the interaction between activated platelets and CTCs to inhibit their metastasis have also yielded promising results. For example, a fibrinolytic platelet system has been developed by loading tissue-type plasminogen activator (tPA) onto platelets to break down CTC aggregates, thereby reducing their immune resistance and metastatic potential (134). Meanwhile, strategies to reduce platelet-tumor cell adhesion by blocking specific signaling pathways, such as the thromboxane A2 (TXA2) prostanoid receptor, have also demonstrated the potential to reduce metastasis in triple-negative breast cancer models (135).

In the realm of predictive modeling, transcriptomic analyses have identified platelet activation-related gene signatures as potential biomarkers for ICI response. A prognostic model constructed using seven platelet activation-associated lncRNAs successfully stratified patients by their likelihood of benefiting from ICIs (42). Likewise, Chen et al. demonstrated that genes involved in the glycoprotein VI-mediated platelet activation (GMPA) pathway are strongly associated with immunotherapy outcomes. A scoring system based on GMPA-related genes showed robust predictive accuracy in assessing ICI efficacy across multiple cancer types (136). Nevertheless, as mentioned earlier, these predictive models based on nucleic acids associated with platelet activation are also currently constrained by limitations such as small sample sizes, tumor heterogeneity, and confounding variables within patient populations. Consequently, these models are not yet suitable for widespread clinical application. Future advancements will necessitate the integration of these models with additional biomarkers, the establishment of standardized testing protocols, and the execution of large-scale, multicenter clinical validations. These steps are essential to facilitate clinical translation and enhance the optimization of treatment regimens involving ICIs.

Taken together, these findings underscore that platelet activation not only orchestrates the dynamic interplay between tumor cells, immune cells, and the vasculature, but also holds diagnostic and therapeutic potential in the era of immunotherapy. However, direct modulation of platelet activation *in vivo* remains challenging due to the risk of disrupting physiological hemostasis. To effectively address these challenges, it is imperative to first establish a standardized protocol for platelet activation assays. Previous research has demonstrated that stable and reliable results can be achieved by meticulously controlling sample storage conditions and employing prepared and stored platelet activation kits (137). Additionally, other studies have indicated that utilizing platelet-monocyte aggregates as a marker of platelet activation can enhance the reproducibility of these assays (138). The development of optimized platelet activation testing protocols is essential not only for advancing our understanding of platelet function in cancer patients but also for facilitating the creation of safe and effective clinical treatment strategies. Furthermore, it is crucial to acknowledge that various factors, including genetic differences, the heterogeneity of tumor biology, and variations in platelet function among patients, can contribute to significant individual variability in cancer patients’ responses to platelet-targeted interventions. These differences can affect treatment efficacy and safety profiles, thereby necessitating the implementation of personalized treatment regimens. In addition, strategies that do not interfere with the physiological mechanisms of normal platelets and that specifically target and block tumor-induced platelet aggregation at the tumor site hold greater promise. One such strategy involves the use of platelet decoys, which are modified platelets that retain binding functions but lack the capacity for activation and aggregation. These decoys can inhibit platelet aggregation and adhesion on thrombogenic surfaces and interfere with platelet-mediated tumor cell aggregation, thereby reducing cancer cell arrest and extravasation (139). Another innovative strategy targets the glycoprotein VI (GPVI) receptor, which is exclusively expressed on platelets and megakaryocytes. Blocking this receptor can disrupt the platelet-tumor cell axis, thereby inhibiting platelet activation by tumor cell-expressed galectin-3 without affecting physiological hemostatic mechanisms (140). These strategies underscore the potential of targeted interventions, thereby advancing the development of safer and more effective antiplatelet therapies. In addition, building on mechanistic insights into platelet activation dynamics and microparticle release, recent advances have shifted attention toward leveraging platelets as functional carriers and therapeutic tools. The following section summarizes the emerging strategies and applications of engineered platelets in enhancing the efficacy and specificity of tumor immunotherapy.

## 6 Engineering platelet: transforming natural carriers into precision immunotherapeutic platforms

Building upon the dual role of platelets as both modulators and messengers within the TME, recent advances in bioengineering have sought to reprogram or repurpose platelets into active participants in cancer therapy rather than passive contributors to tumor progression. Given their intrinsic tumor-homing ability, biocompatibility, and dynamic interactions with immune and vascular systems, platelets offer an exceptional foundation for the development of targeted drug delivery systems and next-generation immunotherapeutic platforms.

### 6.1 Biological basis for platelet engineering

Under physiological conditions, platelets selectively migrate and accumulate at sites of inflammation or vascular injury, guided by chemotactic gradients and adhesion molecule interactions, where they become activated and release cytoplasmic granules rich in cytokines, chemokines, and growth factors (141). Tumors, often conceptualized as “chronic non-healing wounds”, share extensive pathological parallels with inflammatory and reparative processes (90, 142). This similarity endows platelets with a natural affinity for tumor tissues. This process is thought to arise primarily from direct or indirect interactions between tumor cells and platelets, leading to platelet activation and the subsequent release of granules containing adenosine diphosphate (ADP), TXA2, and other substances, which in turn promote platelet aggregation (143).

Furthermore, their micron-scale size, deformability, and ability to traverse dense stromal barriers enable platelets to infiltrate deep tumor parenchyma and engage in intimate crosstalk with malignant and immune cells (144). As discussed in previous sections, these interactions not only regulate immune evasion and ICI responsiveness but also facilitate granule-based molecular exchange. Exploiting this unique spatial and functional specificity, platelet engineering has emerged as a promising avenue for tumor-targeted therapeutic delivery and immune modulation.

### 6.2 Platelet-based delivery systems

**Figure 4:**
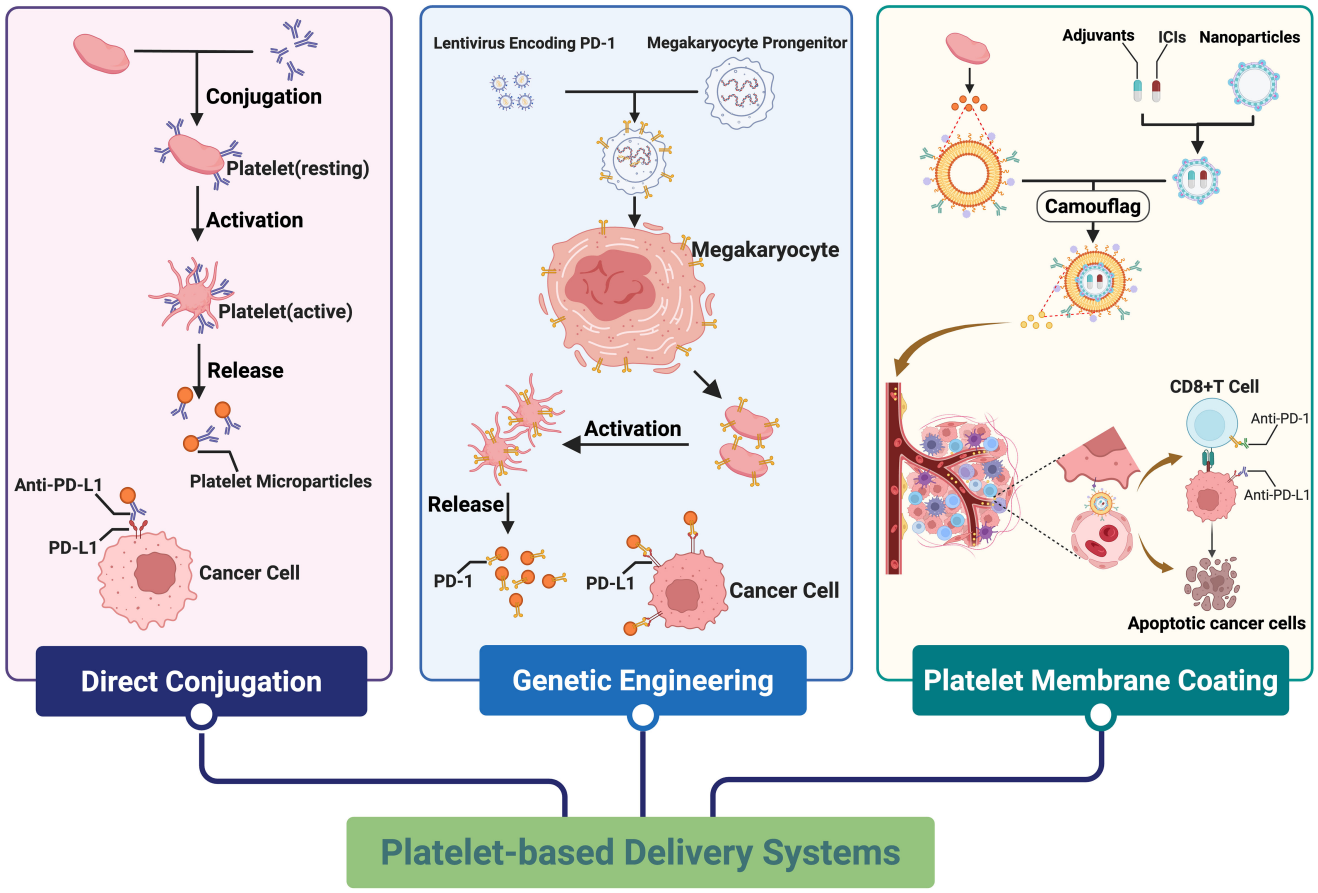
Constructing delivery systems based on platelet function to enhance tumor immune checkpoint inhibitor therapy. Created in https://BioRender.com.

Building on these biological and physical properties, researchers have proposed the concept of platelet reverse engineering, which aims to remodel platelet functions and transform them into precise tumor-targeted drug delivery systems (145). To date, platelet-based delivery systems have evolved along three major approaches, as illustrated in **Figure 4**.

#### 6.2.1 Conjugation of ICIs to platelets

The first approach involves the direct conjugation of ICIs to the platelet surface. For instance, researchers have activated anti-PD-L1 antibodies using the crosslinker Sulfo-SMCC and subsequently incubated them with Traut’s reagent-modified platelets to obtain aPD-L1-decorated platelets (145). Upon entering the tumor site, these platelets become activated and release platelet-derived microparticles (PMPs) carrying the therapeutic antibodies, which locally block PD-L1 on tumor cells. This localized inhibition amplifies cytotoxic T-cell activity while minimizing systemic exposure and off-target toxicity.

#### 6.2.2 Genetically engineered platelets

A second strategy focuses on genetic modification of megakaryocyte progenitors, enabling mature platelets to express therapeutic proteins or receptors. By introducing the PD-1 gene into megakaryocytic precursors, researchers have successfully generated platelets that constitutively express PD-1 on their surface (146). Upon activation within the TME, these engineered platelets release PD-1-bearing PMPs that bind to tumor PD-L1, thereby locally disrupting the PD-1/PD-L1 immune checkpoint axis and potentiating antitumor immunity. This genetic approach exemplifies the potential of platelets as living carriers that actively engage immune signaling pathways to enhance therapeutic specificity.

#### 6.2.3 Platelet membrane-based nanocarrier systems

The third and most versatile strategy integrates nanotechnology with biomimetic engineering through the creation of PM-coated nanoparticles. These systems capitalize on the functional proteins and receptors preserved in the PM to maintain platelet-like targeting and immune evasion properties (147). This platform not only improves targeting precision and therapeutic efficacy but also enhances nanocarrier stability and biocompatibility, reducing aggregation caused by serum protein adsorption, vascular occlusion, or nonspecific adhesion. Moreover, the membrane coating effectively prevents rapid immune clearance mediated by the mononuclear phagocyte system (148, 149).

In the realm of cancer immunotherapy, PM-based nanoplatforms have been utilized to co-deliver ICIs, chemotherapeutics, and targeted agents in a spatiotemporally controlled manner. For instance, Da et al. designed PM-coated hollow mesoporous silica nanoparticles co-loaded with sorafenib and PD-1 inhibitors, achieving precise tumor targeting and significant antitumor efficacy in hepatocellular carcinoma models (150). Comparable PM-based nanocomposites, such as NK cell-driven delivery systems and photothermal therapeutic platforms, have also demonstrated potent synergistic effects (43, 151). Notably, these multifunctional PM-coated nanoplatforms enable the co-delivery of immunotherapeutic agents, targeted drugs, and chemotherapeutics, addressing the current need for combination regimens that yield enhanced and synergistic antitumor effects (152). Furthermore, their high targeting specificity effectively reduces the off-target binding of ICIs to normal tissues, thereby enhancing therapeutic efficacy while mitigating irAEs (153).

### 6.3 Strategies to enhance tumor-targeting capability of platelets

To further improve platelet homing and retention within tumors, ligand-based molecular engineering has been employed to enhance the affinity of engineered platelets for tumor vasculature and malignant cells. Researchers have identified and engineered a fusion protein composed of the extracellular domain of tissue factor (truncated tissue factor, tTF) and a GNGRAHA peptide (154). This fusion protein specifically targets tumor vascular endothelial cells and induces localized thrombosis, thereby increasing platelet recruitment and accumulation within tumor tissue (155). Based on the same principle, Wang et al. developed a fusion protein consisting of tTF and an Arg-Gly-Asp peptide, which selectively binds to tumor neovasculature and activates the coagulation cascade. This process facilitates the accumulation of anti-PD-1 antibody-conjugated platelets within tumor tissues and leads to significant antitumor efficacy in a breast cancer model (147).

Meanwhile, to shorten the spatial distance between tumor cells and T cells and thereby potentiate T cell-mediated cytotoxicity, Chen et al. designed anti-CD3 modified platelets that can simultaneously anchor to both tumor cells and T cells (156). This dual-anchoring mechanism effectively promotes immune synapse formation, resulting in enhanced inhibition of tumor recurrence and metastasis.

Collectively, despite these remarkable advancements, most engineered platelet systems remain confined to preclinical stage, including *in vitro* investigations and animal models. Significant challenges must still be addressed before these platforms can progress to clinical trials, achieve successful clinical translation, and ultimately be implemented at scale to benefit patients with malignancies.

During the design and synthesis of platelet-derived delivery systems, biosafety considerations are of paramount importance. The use of autologous platelets or platelets obtained from matched healthy donors each presents distinct advantages and limitations, particularly with respect to interpatient variability, platelet heterogeneity, and the feasibility of large-scale clinical application. In this context, the expansion and differentiation capacity of induced pluripotent stem cells may represent a promising alternative strategy to overcome the quantitative constraints associated with platelet sourcing and large-scale production. From cell preparation and ex vivo manipulation to the final construction of the drug delivery platform, strict control is required to ensure the absence of chemical or biological contaminants throughout the manufacturing process. Concurrently, continuous optimization of drug-loading efficiency, cargo stability, and target-site drug release kinetics is essential. Equally important is the simplification and standardization of the manufacturing workflow, which would improve process reproducibility, facilitate large-scale production, and minimize batch-to-batch variability. Moreover, preserving the functional integrity of platelets after engineering modification, together with maintaining favorable biocompatibility, is critical for ensuring the *in vivo* stability, pharmacokinetic performance, and precise targeting capability of the delivery system, thereby maximizing therapeutic efficacy. Another important limitation is that most currently reported platelet-based delivery systems are administered shortly after synthesis, which restricts their practical clinical applicability. Therefore, improving the stability of these engineered platforms during storage and transportation will be essential for enabling broader clinical use. In addition, the complexity of the tumor microenvironment, including intertumoral diversity across cancer types, intratumoral heterogeneity, and interpatient variability, must be carefully considered when evaluating the therapeutic potential of such systems. As mentioned previously, responses to ICIs vary substantially among patients, and the overall response rates in many tumor types remain relatively modest. Consequently, even with the potential therapeutic enhancement provided by engineered platelet systems, the associated increase in treatment costs highlights the importance of identifying robust predictive biomarkers that can accurately stratify patients and guide the selection of optimal ICIs-based therapeutic strategies.

Despite these challenges, the broad translational potential and anticipated therapeutic benefits of engineered platelet technologies remain highly compelling. With continued advances in biomaterials engineering, cell engineering, and precision immunotherapy, many of the current limitations are likely to be progressively addressed. Ultimately, this emerging strategy may transform platelets from passive facilitators of tumor progression into active mediators of antitumor immune responses, thereby opening new avenues for the development of more precise, effective, and safer cancer therapies.

## 7 Conclusion and prospective

In summary, exploring the role of platelets in ICI therapy has become a research frontier in the field of tumor immunology. Platelets have emerged as multifunctional regulators within the TME, exerting profound influences on immune modulation, tumor progression, and therapeutic responsiveness. Beyond their classical roles in hemostasis, platelets actively shape antitumor immunity through the expression and transfer of immune checkpoint molecules, the delivery of nucleic acids, and dynamic interactions with tumor and immune cells.

In tumor immune assessment, based on the interaction mechanisms between platelets and tumor cells as well as other immune cells, the development of platelet-related proteins, such as PD-L1, can be used for early screening of tumors and assessment of the efficacy of ICIs. Other platelet-associated biomarkers, including platelet counts, activation status, platelet-derived nucleic acids, and PRGs signatures, have also demonstrated considerable predictive value for both the efficacy of ICIs and the risk of irAEs. These biomarkers provide accessible and cost-effective tools for real-time immunomonitoring and prognosis. Compared with the traditional tumor pathology biopsy, platelet-related biomarkers to assess the tumor immune microenvironment have the advantages of being non-invasive, reproducible and more reflective of the overall situation. Meanwhile, large-scale clinical studies are needed to further validate the predictive value of these markers, which is important for optimizing treatment options.

In tumor immunotherapy, a precise therapeutic system based on the platelet-associated immune-regulatory network is established through targeted regulation of platelet-derived signaling pathways within tumor immune responses. This strategy utilizes function-selective modification technology to systematically reprogram the multidimensional ecosystem of the tumor immune microenvironment under the premise of maintaining the functional integrity of platelet homeostasis, thereby significantly enhancing the anti-tumor efficacy of ICIs and ultimately achieving the elimination of malignant cells. Meanwhile, recent advances in platelet engineering further highlight the translational potential of these anucleate fragments. By harnessing their intrinsic tumor-homing ability, granule-mediated cargo release, and immune-compatible surface properties, platelets can be reprogrammed as precise drug delivery vehicles or functional immunomodulators. Strategies such as conjugation with ICIs, genetic modification, and membrane-based nanocarriers have demonstrated potent antitumor effects in preclinical models, providing a compelling rationale for integration into next-generation combination immunotherapy regimens.

Despite notable advances in delineating the complex interplay between platelets, tumor cells, and immune constituents, current insights capture only a fraction of the profound and multifactorial roles platelets exert across tumor initiation, progression, and therapeutic response. Substantial mechanistic ambiguities and translational constraints continue to impede the full realization of platelet-centered clinical applications. Moving forward, a critical research priority lies in dissecting the immunoregulatory circuits through which platelets influence antitumor immunity, with a particular focus on the mechanisms governing platelet-mediated tumor immune checkpoint molecules transfer and the modulation of immune effector cell phenotypes. Elucidating these pathways will not only refine our conceptual framework of platelet biology in cancer but also unveil previously unrecognized therapeutic vulnerabilities. Concurrently, the integration of high-resolution multi-omics technologies, next-generation bioengineering platforms, and systematically designed clinical trials is anticipated to facilitate the classification of platelets into well-defined subpopulations with distinct functional profiles. This advancement is expected to transform platelets from merely informative biomarkers or accessory modulators into genuine, actively engineered therapeutic entities, thereby advancing the precision, robustness, and translational impact of cancer immunotherapy.

## Glossary

ICIs: immune checkpoint inhibitors
PD-1: programmed cell death protein 1
PD-L1: programmed cell death protein ligand 1
CTLA-4: cytotoxic T-lymphocyte-associated protein 4
irAEs: immune-related adverse events
TME: tumor microenvironment
PM: platelet membrane
VEGF: vascular endothelial growth factor
TGF-β: transforming growth factor β
IHC: immunohistochemistry
miRNAs: microRNAs
ctDNA: circulating tumor DNA
CTCs: circulating tumor cells
TEPs: tumor-educated platelets
TPH: tryptophan hydroxylase
PFS: progression-free survival
ORR: objective response rate
RGS18: regulator of G-protein signaling 18
HMGB1: high-mobility group box1
mRNAs: messenger RNAs
circRNAs: circular RNAs
lncRNAs: long noncoding RNAs
PMPs: platelet-derived microparticles
PRGs: platelet-related genes
HDL: high-density lipoprotein
TPS: tumor proportion scores
PR: partial response
PRS: platelet risk scores
TUBA1C: alpha1C-tubulin
PLR: platelet-to-lymphocyte ratio
OS: overall survival
NSCLC: non-small cell lung cancer
sEVs: small extracellular vesicles
EMT: epithelial-mesenchymal transition
MCP-1: macrophage chemotactic protein-1
IL-1α: interleukin-1α
SDF-1: stromal cell-derived factor-1
HIF-1: hypoxia-inducible factor 1
MHC: major histocompatibility complex
PDGFs: platelet-derived growth factors
PFTBA@Alb: albumin-based perfluorotributylamine nanoparticles
tPA: tissue-type plasminogen activator
TXA2: thromboxane A2
GMPA: glycoprotein VI-mediated platelet activation
GPVI: glycoprotein VI
ADP: adenosine diphosphate
tTF: truncated tissue factor.
